# A “Bat” Is Easier to Learn than a “Tab”: Effects of Relative Phonotactic Frequency on Infant Word Learning

**DOI:** 10.1371/journal.pone.0059601

**Published:** 2013-03-20

**Authors:** Nayeli Gonzalez-Gomez, Silvana Poltrock, Thierry Nazzi

**Affiliations:** 1 Université Paris Descartes, Sorbonne Paris Cité, Paris, France; 2 CNRS, Laboratoire Psychologie de la Perception, Paris, France; University of California, Davis, United States of America

## Abstract

Many studies have shown that during the first year of life infants start learning the prosodic, phonetic and phonotactic properties of their native language. In parallel, infants start associating sound sequences with semantic representations. However, the question of how these two processes interact remains largely unknown. The current study explores whether (and when) the relative phonotactic probability of a sound sequence in the native language has an impact on infants’ word learning. We exploit the fact that Labial-Coronal (LC) words are more frequent than Coronal-Labial (CL) words in French, and that French-learning infants prefer LC over CL sequences at 10 months of age, to explore the possibility that LC structures might be learned more easily and thus at an earlier age than CL structures. Eye movements of French-learning 14- and 16-month-olds were recorded while they watched animated cartoons in a word learning task. The experiment involved four trials testing LC sequences and four trials testing CL sequences. Our data reveal that 16-month-olds were able to learn the LC and CL words, while14-month-olds were only able to learn the LC words, which are the words with the more frequent phonotactic pattern. The present results provide evidence that infants’ knowledge of their native language phonotactic patterns influences their word learning: Words with a frequent phonotactic structure could be acquired at an earlier age than those with a lower probability. Developmental changes are discussed and integrated with previous findings.

## Introduction

In the past decades a large number of studies have focused on exploring how infants’ speech perception abilities become tuned to their native language and how infants start associating sound sequences with semantic representations (i.e., learning words). Traditionally, these two learning processes have mostly been explored independently from each other and only recently has the interaction between phonological and lexical development received increased attention (see [1] for a review focusing on language production). The present study aims to investigate a potential link between perceptual acquisition and early word learning. More specifically, it explores whether (and when) the relative phonotactic probability of a sound sequence in the native language has an impact on infants’ word learning. Phonotactics corresponds to “constraints on the possible sequencing and positions of phonetic segments allowed in words” ([2] p. 631). For each language, a phonetic sequence can either be phonetically legal, or illegal (e.g.,/kn/is legal in syllable onset in Dutch but not in English). Moreover, within phonotactically legal sequences, some can be more typical/probable, while others can be legal yet atypical/less probable (e.g., for English, high-probability/kæz/versus low-probability/gu∫/), based on differences in the frequency of its constituting phonemes and phoneme sequences. It is this relative phonotactic distinction that will be investigated here.

Before infants are able to learn words, they have to deal with a huge amount of information in order to discover the relevant phonological properties of their native language, and learn its prosodic, phonetic, and phonotactic characteristics (e.g., [2]–[8]). This learning starts as soon as the second half of the first year of life. For example, before 6 months of age, infants are able to discriminate both native and nonnative phoneme contrasts, but this discrimination ability is shaped by the native phonological system by 6 months for vowels and by 10–12 months for consonants [3], [7]–[8]. Similarly, infants’ attunement to the prosodic characteristics of the native language is illustrated by the finding that English- and German-learning infants start preferring to listen to words with a trochaic (strong-weak) stress pattern over words with an iambic (weak-strong) stress pattern, the former being more frequent in both languages, by 9 and 6 months respectively [5], [9].

Concerning phonotactic acquisition, different studies have shown that before their first birthday, infants are sensitive to the phonotactic properties of their native language. For example, 9-month-old infants are able to distinguish between legal and illegal sequences in their native language, and show a preference for legal sequences [4], [6], [10]. Around the same age, infants were also found to be sensitive to the overall frequency of some phonemes or the frequency with which phonotactically legal sequences appear in the words of their language, preferring the more frequent over the less frequent sequences [2], [11]–[12].

In parallel to phonological acquisition, infants become able to map sounds to meaning. Some beginnings of word comprehension have been found as early as 6 months of age, when infants show evidence of comprehending very frequent words like “daddy” and “mommy,” or “hand” and “feet” [13]–[14]; see also [15]. By 8 months, infants are able to associate novel words to their referent objects when the object’s movement is coherent with word presentation [16]. By 12 months, word learning is possible if supported by social cues (i.e. eye gaze, [17]) and by 14–16 months, even in the absence of social cues [18]–[19], or when using similar-sounding words [20]–[21].

The results cited above clearly demonstrate that infants are able to detect phonotactic patterns in their native language on the one hand, and to map sounds with meanings by their first birthday on the other hand. Nevertheless little is known about whether this phonotactic knowledge learned during the first year of life constrains early lexical acquisition.

There is some evidence showing that phonotactic knowledge can affect word learning both in children and adults. Different studies have shown that 3-to-13-year-old children could learn novel words more readily when labels contained frequent sound sequences than when labels contained infrequent sound sequences, a distinction based on phone and biphone positional frequency [22]–[24]. Furthermore, phonotactic high-probability pseudo-words have been found to be repeated more accurately [25]–[26] and to be better recalled [27] than low-probability pseudo-words in 3- to 8-year-old children. Likewise these effects have also been found in adults, pseudo-words with a frequent phonotactic structure being repeated faster [28]–[30], and rated to be more word-like [31]–[33] than low-probability pseudo-words.

Although we find these phonotactic effects on word learning and word processing in older children and adults, this does not necessarily mean that this is the case during infancy. Different studies have shown that infants are more flexible than children or adults in the range of forms that they are ready to accept as object labels. Specifically, young infants have been found to accept words, gestures, non-verbal sounds, and pictograms as labels for objects, and that the scope of accepted labels narrows down during the second year of life [17], [34]–[38].

In this context, a few studies have addressed the question of the existence of phonotactic constraints on early word acquisition. Some of the research on this topic has focused on infants’ early word production [39]–[40], showing that by 2 years of age, toddlers can repeat pseudo-words’ segments with a high phonotactic probability more accurately both in onset and coda positions. To the best of our knowledge only two recent studies investigated the relation existing between infants’ ability to map sound sequences with their referents and the phonotactic probability of a sound [41]–[42]. Graf Estes and colleagues [41] tested 17-to-20-month-old English-learning infants with two novel object labels being either phonotactically legal (e.g., *dref*) or illegal in English (e.g., *dlef*). These infants readily learned the word-object pairings in the phonotactically legal condition, but had difficulties in learning the illegal labels. Additionally, the authors found that the link that exists between phonotactic knowledge and word learning correlated with vocabulary size: the larger the receptive vocabulary, the greater the difference between performance in learning legal and illegal labels.

Meanwhile, MacKenzie and colleagues [42] tested English-learning 12-month-olds’ ability to associate different types of words with novel objects: novel English words, Japanese words (phonotactically legal in English, but phonetically different), and Czech words (violating English phonotactics). In this study, infants were able to associate the novel objects with the English and the Japanese words but not the Czech ones. Thus, English-learning infants were able to map words having the legal phonotactic properties of English.

Taken together, the results of Graf Estes et al. [41] and MacKenzie et al. [42] show that infants’ language-specific knowledge about phonotactic patterns can have an influence on word acquisition early in life (by 12 months according to the results of [42]; and by 20 months of age as suggested by the results of [41]). However, given that both studies contrasted phonotactically legal versus illegal labels, the scope of these constraints is not clear, and whether the effects observed would hold for relative phonotactic frequency remains unclear. Indeed, while it is possible that illegal sequences are processed as legal words with null phonotactic frequencies, which would be consistent with a relative frequency interpretation of the phonotactic patterns observed, a second possibility is that legal and illegal sequences may not be processed in the same way, and that illegal sequences are being processed as non-native signals. In this perspective, the previous findings do not tell us anything about infants’ sensitivity to relative phonotactic frequency while learning new words. In order to show this, one needs to explore phonotactic effects on early word learning by comparing legal sequences that differ in their relative phonotactic frequency.

This issue of the effects of relative frequency has recently started to be explored in both the prosodic and phonotactic domains. For prosody, Floccia, Nazzi, Austin, Arreckx and Goslin [43] tested whether English-learning 20–24-month-olds would learn trochaic nouns for objects more easily than iambic nouns, the former being the predominant stress pattern of bisyllabic English nouns (about 90% trochaic); their results failed to find differences in acquisition performance. However, Curtin, Campbell and Hufnagle [44] explored a similar issue, but with younger infants (English-learning 16-month-olds) and regarding the acquisition of verbs rather than nouns, disyllabic verbs being mostly iambic in English (about 70% iambic). They found that infants could learn the iambic labels for the verbs but not the trochaic ones. Since both trochaic and iambic labels are legal English verbs, the difference in performance established effects of the relative typicality/probability of sound patterns in the prosodic domain.

Concerning phonotactics, Hollich, Jusczyk and Luce [45] manipulated in the laboratory the phonotactic frequency of a target word (e.g., tirb) by familiarizing 17-month-olds with 12 words and manipulating the number of these words that were phonotactically related to the target (e.g., tirsh, lirb…): 12 in one condition, only 3 in the other condition. Then, they conducted a classic word learning task using the preferential looking paradigm. At 17 months of age, infants succeeded in learning the words only if they had been familiarized with twelve phonotactically-related words, showing that familiarity to a phonotactic pattern facilitates word learning. In this study, however, phonotactic probability was manipulated experimentally in the laboratory (by varying the amount of co-occurrences between the phonemes, and using the same phonemes in the target and related words), which could restrict the generalization of the findings.

In the present study, we investigate the role that the phonotactic knowledge about the native language acquired in the first months of life in the infant environment could play when learning new words at the onset of lexical acquisition. Our goal is thus to explore whether (and if so, when) the relative phonotactic probability of a sound sequence in the native language has an impact on infants’ word learning. To investigate this question, we exploit the fact that Labial-Coronal (LC) words are more frequent than Coronal-Labial (CL) words in early word production and in the lexicon of many languages.

In early word production studies, it has been found that during the 50-word-stage English- and French-learning infants tend to produce more Labial-Coronal (LC) words such as “bat” (i.e., words starting with a labial consonant followed by a coronal consonant) than Coronal-Labial (CL) words such as “tab” (i.e., words starting with a coronal consonant followed by a labial consonant). This Labial-Coronal bias has first been interpreted in terms of production constraints according to which producing an LC sequence requires less and easier movements than producing a CL sequence [46]–[47].

However, it has also been shown that in French, the language of the infants tested in the present study, LC words are more frequent than CL words (with a 63%–37% ratio in all words and a 85%–15% ratio in CVC words only, [11]). A study by MacNeilage and colleagues [48] suggested that such an LC bias might be predominant in the languages of the world, as it was found in 8 out of the 10 languages from different linguistic families they investigated (English, Estonian, French, German, Hebrew, Maon, Quichua, and Spanish). However, there is also some evidence that this pattern is not universal. First, it was not found in Japanese and Swahili ([48]; see however [49], for more nuanced data on Japanese). Second, a more detailed analysis of the relative frequencies in the French lexicon revealed that even if the LC bias is present for plosive and nasal sequences, it is not the case for fricative sequences for which the opposite CL bias was found (c.f. [50]). These variations in bias both across and within languages suggest that it might be acquired in development.

Two recent perceptual studies have investigated whether French-learning infants are sensitive to the relative frequency of LC and CL words in their native language. These studies found that infants start preferring to listen to the LC words between 6–7 and 10 months [11]–[12]. These results indicate that by 10 months of age French-learning infants have already learned that LC sequences are more frequent than CL sequences in French. However, Japanese-learning 7- and 10-month-old infants show neither a preference for LC sequences, nor a preference for CL structures [51]. These cross-linguistic differences are predicted by the properties of the lexicon of the languages contrasted. In accordance with these results, it appears that exposure to the linguistic input is a key factor in the emergence (or not) of the LC bias. These results are in line with all the data showing that during the first year of life infants become increasingly tuned to the characteristics of their native language [5], [7]–[9]. Importantly, Gonzalez-Gomez and Nazzi [11] showed that 10-month-olds do not show any preference when they are presented to the CV or VC sequences making up the original LC and CL stimuli. These results rule out interpretations of the LC preference in terms of adjacent dependencies, or positional frequencies such as the existence of L-initial or C-final biases (note that Coronal-initial and -final sequences are both more frequent than words with a labial consonant in either onset or coda position in French, c.f. [11]). On the contrary, they support an interpretation of the LC bias as a preference for the relative position of the non-adjacent consonants.

The predominance of the LC structures in the lexicon and the early listening preference found for these sequences in French-learning infants makes the LC bias a good candidate to explore how phonotactic probability of a sound sequence in the native language might influence infants’ word learning. We predict that LC words will be learned more easily and thus at an earlier age than CL sequences. This prediction is based on the fact that, as suggested by Saffran and Graf Estes [52], high-probability sequences are composed by very familiar sound combinations, which are phoneme sequences that infants might have experienced many times. This familiarity may decrease the computational load in word learning situations, a hypothesis referred to as the “encoding-facilitation” effect (which is compatible with findings that pre-exposure to either a word form or an object enhances word-object mapping, [53]–[54]). If high-probability sequences are easier to encode and remember, then infants can dedicate more computational resources to mapping sounds with meaning when learning a high-probability new word. On the contrary, when learning low-probability new words, they will need more cognitive resources to encode the sound sequence, which will make linking the sound sequences to their meaning more difficult. In other words, “easily-acquired and early learned words may tend to consist of high-probability sound sequences” ([52], p.35) such as LC words. This interpretation would be compatible with the results of Graf Estes et al. [41] and MacKenzie and colleagues [42]. However, their evidence is limited to an advantage for legal over illegal words, which could be processed qualitatively differently than both high and low probability words as discussed earlier.

In a previous study by Nazzi and Bertoncini [55], no difference between learning LC and CL words was found in 20-month-old French-learning infants. That study used the name-based categorization task [56] in which triads of unfamiliar objects are presented. For each triad, two objects were labeled with the same name and the third object was labeled using a different name. Only minimal pairs of words were used (i.e. LC/pid/and/pit/, or CL/dap/and/tap/). The authors offered different explanations for this null result. The first one is that phonotactic regularities do have an impact on word learning but that this effect is developmentally transient, and that the infants tested were already too old. The second one is that the task they used was not sensitive enough to show such differences. In order to continue the exploration of such effects, we used in the present study a multi-trial cartoon-learning task that only presented two objects per trial, with no minimal pairs, to make the task easier. In addition, we used an eye-tracker to record infants’ eye movements (similarly to what was done by [41]) that allows us to analyze the looking behavior instead of the explicit reaching behavior of the infants. Additionally, we tested younger infants to explore potential developmental differences.

In Experiment 1, we focus on 16-month-old infants because we know that infants from the age of 14 months on are able to associate two different objects with dissimilar sounding words (e.g., *neem* and *lif*; [57]–[58]) or even similar sounding words (e.g., *bin* and *din*; [21]) in laboratory tasks. Besides, as infants at 16 months display a large amount of variability in their receptive vocabulary (for English-acquiring infants, [59]), this allowed to test if vocabulary size is related with learning words of different phonotactic probabilities as has been shown by Graf Estes et al. [41].

## Experiment 1

### Materials and Methods

#### Ethics statement

Parents of all infant participants provided written informed consent prior to the experiment. The experimental protocol and consent procedure were both approved by the CERES (Comité d’évaluation éthique des projets de recherche) of the Université Paris Descartes. All data were obtained according to the principles expressed in the Declaration of Helsinki.

#### Participants

Twenty-eight full-term 16-month-old infants from French-speaking families were tested and included in the analyses (mean age = 16 months 9 days; range: 16 months 1 day –16 months 23 days; 14 girls, 14 boys). Fifteen additional infants were tested and excluded from the analyses due to fussiness/crying (N = 3), technical problems (N = 1) or because they did not meet the inclusion criteria (N = 11; see paragraph on data analysis for details). The age of the rejected infants did not differ from the age of the included infants (mean age = 16 months 11 days, range: 16 months 4 days –16 months 23 days), and both female (N = 11) and male (N = 4) participants were excluded.

#### Stimuli

Speech Stimuli. The speech stimuli consisted of 8 pairs of monosyllabic C_1_VC_2_ pseudo-words or low frequency words not likely to be known by infants (see Table 1). Half of them involved labial-coronal (LC) structures and the other half coronal-labial (CL) structures. Items in both conditions were made up of exactly the same plosive consonants (/p/,/b/,/d/,/t/) and vowels (/⊂/,/œ/,/u/,/i/). Vowels had been chosen in order to obtain balanced adjacent biphone and triphone frequencies between the LC and CL lists for the C_1_V, VC_2_ and C_1_VC_2_ sequences of phonemes (C_1_V *t*_(15) = _1.15; *p* = .33, VC_2_ *t*_(15)_ = 0.48; *p* = .66, and C_1_VC_2_ *t*_(15) = _0.81 *p* = .44) according to the Lexique 3 database [60]. Therefore, while in Saffran and Graf Estes [52] “high probability” sequences were defined in terms of adjacent phonemes, in our experiment all the adjacent frequencies were fully controlled, so that the only difference between the two lists of items used here was the overall relative frequency for the LC and CL non-adjacent sequences in the French lexicon. All items were recorded in a sound-attenuated booth by a female French native speaker. The duration for the LC and the CL pseudo-words was similar (386 vs. 375 ms, *t*_(127) = _1.34; *p* = .22).

**Table 1:** Pairs of Labial-Coronal and Coronal-Labial CVC sequences used in Experiments 1 and 2.

	Labial-Coronal pairs			Coronal-Labial pairs		
	Word/Pseudo-word_1_	Word/Pseudo-word_2_		Word/Pseudo-word_1_		Word/Pseudo-word_2_
**Pair_LC 1_**	bode/b⊂d/	peute/pœt/	**Pair_CL 1_**	dibe/dib/	teupe/tœp/	
**Pair_LC 2_**	bide/bid/	poute/put/	**Pair_CL 2_**	daube/d⊂b/	toupe/tup/	
**Pair_LC 3_**	bote/b⊂t/	peude/pœd/	**Pair_CL 3_**	doupe/dup/	teube/tœb/	
**Pair_LC 4_**	boute/but/	pid/pid/	**Pair_CL 4_**	dope/d⊂p/	tibe/tib/	

**Figure 1:**
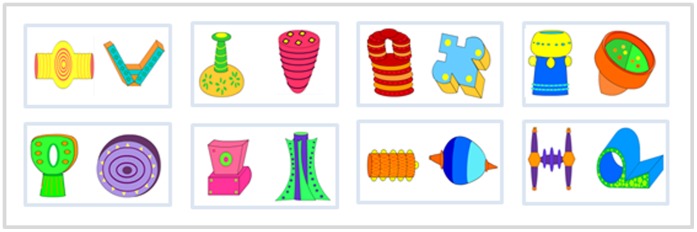
Object stimuli. Pairs of novel objects used in Experiments 1 and 2.

Object Stimuli. Images of eight pairs of objects differing in shape, color and texture (Fig. 1) were created for the current study. The reason for using clearly different objects and clearly different words was to facilitate learning of the word-object pairings. All objects were selected so that children and adults would be unfamiliar with them. The object pairs were consistently associated with one pair of LC words and one pair of CL words, presented to different infants.

**Figure 2:**
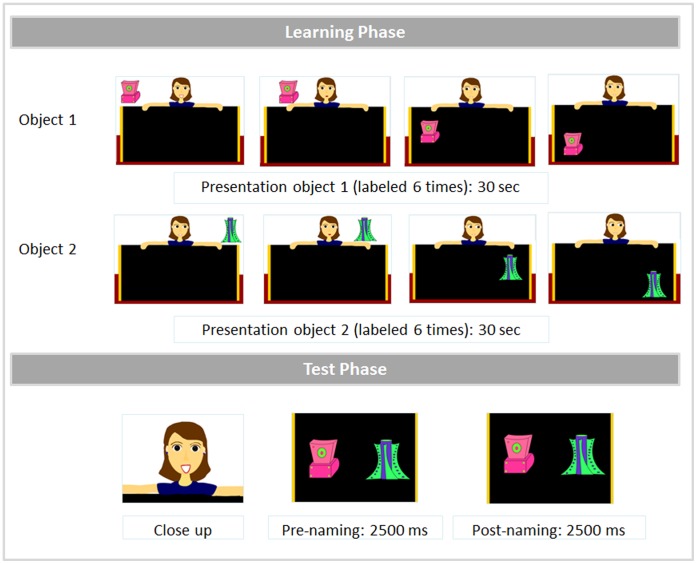
Structure of a word-learning cartoon.

Cartoons. The word-object pairings were embedded into word-learning cartoons, using Adobe Flash. In each trial, a female character behind a black board presented the two objects, one at a time (Fig. 2, learning phase). The first object always appeared in the left upper corner of the screen. At the beginning, the object moved horizontally in the upper left part of the display, while it was labeled three times (Look! A [target]! This is a [target]. Look at what I’m going to do with the [target]!). Then, the object started shifting down, while it was labeled one more time (I put the [target] here). It started moving vertically in the lower left part of the screen and was labeled two more times (Have you seen the [target]? Look carefully at the [target]!) before disappearing. The second object was always introduced in the upper right corner of the display and followed a trajectory analogous to that of the first object. The cartoon experimenter followed the objects’ movements with her eyes. Participants were successively trained on each label-object pairing for 30 seconds. The entire learning phase lasted 1 minute and each label was repeated 6 times.

After the learning phase, there was a close up on the face of the cartoon experimenter saying: “Look!” in order to direct infants’ fixations to the center of the screen. After the face disappeared, the two objects appeared at the same time, each on the side it appeared during the learning phase, and started moving synchronously in a vertical way, while the out-of-sight speaker said: “Look at the [target]? Where’s the [target]!” (Fig. 2, test phase). The test phase was divided into two parts lasting 2500 ms each (a prenaming phase and a postnaming phase), time-windows that have been shown to cover lexical processes related to word form processing in the second year of life [61]–[64]. The “prenaming phase” served to evaluate any potential spontaneous preference for a given object, prior labeling. The postnaming phase evaluated the recognition of the target object after its label had been pronounced. This phase started 367 ms after the onset of the target word. This value corresponds to the amount of time required to initiate an eye movement in response to an auditory stimulation in 14-to-24-month-olds and it has been used in numerous studies on early lexical processing (e.g., [61]–[64]).

### Apparatus and Procedure

The films were presented on a 17″ TFT monitor (1280×1024 pixel resolution) with an integrated Tobii T60 eyetracking system which was run by a DELL PC computer. A camcorder was mounted above this display to monitor the participants’ behavior. The presentation of the stimuli and the storing of the data were performed with the Tobii Studio software.

Participants were tested individually in a dimly lit, sound-proof laboratory room. Each infant sat approximately 65 cm from the screen on a caregiver’s lap in the center of the test booth. The caregiver was wearing opaque glasses to prevent them from seeing the stimuli and thus minimize the potential for biases. The experimenter controlled the presentation of the stimuli from an adjacent room and monitored the participant’s behavior through a video camera. The session began with a 5-point infant calibration. Then a small animation was displayed on the center of the screen before each of the 8 trials until the infant looked at it, in order to start each trial at the center of the screen.

Each of the 8 trials had the same structure, and corresponded to the presentation of a cartoon. Each trial was thus composed of the learning of 2 LC (e.g., ‘object 1’−/b⊂d/, ‘object 2’−/pœt/) or 2 CL words (e.g., ‘object 1’−/d⊂p/, ‘object 2’−/tib/), immediately followed by a testing phase evaluating learning/recognition. In the test phase of each trial, infants were required to look at one of the two objects (e.g.,/b⊂d/). Therefore, within each trial, one object was the target and the other one was the distractor.

There were eight pseudo-randomized orders counterbalancing for target side, target object, trial order and object label. Thus, between-subjects counter-balancing ensured that each label was presented and tested on the right and left side and each object was labeled with an LC and with a CL word. Within-subjects, the first and the second half of the test trials always contained 2 LC and 2 CL trials (8 trials in total: 4 LC and 4 CL ones), and half of the time the target word was on the left, half of the time it was on the right. None of the objects or words was presented twice during the test. The experiment lasted approximately 10 minutes.

### Data Analysis and Exclusion Criteria

The eye-tracking data used for the analysis consisted of the binocular gaze position (X and Y coordinates) at each timestamp, that is, every 16.6 msec. First, the proportion of on-screen looks during the course of the 8 trials was calculated for each infant. We excluded six infants with less than 50% on-screen data (between 32% and 49%) to ensure that infants were sufficiently engaged in the task (see also [65], for a similar inclusion criteria).

For each trial, we then calculated the proportion of on-screen looks as well as the proportion of time infants spent looking at the target (T) and the distractor (D) in both the prenaming and the postnaming phases. Therefore, two areas of interest were defined (575×895 Pixel), each including one object. Trials in which infants had a strong object bias in the pre-naming phase (>90% looking to one object) and trials with more than 50% missing data were discarded from the analysis (67/264 trials, 25.4% of the trials). This was done in order to remove trials in which the baseline preference was not well established (similar to [66]–[67]), and in which the participant is considered as not sufficiently exposed to the material of the given trial (see also e.g. in [65], [68]). Finally, only those infants who had at least two analyzable trials per condition were included to have a better estimate of each infant’s “true” naming effect (N = 5 did not meet this criterion; for a similar criterion, see also [69]). Although the attrition rate of 35% (15 out of 43 infants) appears rather high, it is not unusual for infant studies using remote eye-trackers (e.g., in [70], testing 9-month-olds: 59%; [71] testing 14-month-olds: 44%; [72] testing 24-month-olds: 42%). In the final sample, each participant provided, on average, 6.35 trials out of 8. During each of the 30-sec learning phase, 16-month-old infants spent 9.4 s on average looking at the object and 12.1 s looking at the woman’s face. There were no difference in looking times at the objects for the LC (9.3 s) and CL (9.6 s) words.

### Vocabulary Measure

To determine the size of the infants’ receptive vocabulary, parents were asked to fill out the vocabulary part of the French equivalent [73] of the MacArthur Communicative Development Inventory: Toddlers (CDI; [74]).

### Results and Discussion

#### Label recognition measure: proportion of target looking

To examine object-label recognition, the *proportion of target looking* in the prenaming and postnaming phases was calculated for each trial by dividing the looking time to the target object by the time spent looking to the distractor and the target (T/(D+T)). For each infant, this measure was then averaged across trials for the two phases (prenaming/postnaming) and for the two conditions (LC/CL) separately, leading to four values per infant. A repeated measures ANOVA with phase (pre- vs. postnaming phase) and condition (LC vs. CL) as within-subject factors, gender (female vs. male) as between-subject factor and proportion of target looking as dependent variable revealed a significant main effect of phase (*F*(1, 26) = 5.08, *p* = .03, *η_p_^2^* = .16 corresponding to an increase in target looking from the prenaming (*M* = 49.44%, *SD* = 13.01%) to the postnaming phase (*M = *54.32%, *SD* = 10.83%). Neither the effect of condition, *F*(1, 26) <1, nor the interaction between condition and phase, *F*(1, 26) <1, reached significance. In addition, no effects including the factor gender reached significance (all *F* <1). Thus, irrespective of condition, 16-month-olds increased their looking toward the object that was labeled after hearing the name of the target.

#### Influence of vocabulary size

Graf Estes et al. [41] reported a positive correlation between target looking and receptive vocabulary size for the phonotactically legal words, and a marginally significant negative correlation for phonotactically illegal words, a pattern which indicates that increasing knowledge about word forms in the native language is related to 17–20-month-old infants’ consideration of a constrained set of sound sequences as possible new words. To evaluate if learning of phonotactically high and low probability sound sequences was modulated by the receptive vocabulary size, correlational analyses were conducted between target looking and the CDI score. Therefore, the mean difference score of target looking between the pre- and postnaming phases ([% target looking in postnaming phase −% target looking prenaming phase]) was calculated for each participant and both structures. For both the LC words and the CL words, there was no significant relationship between object label recognition and receptive vocabulary size (LC condition: *r* = −.01; *p* = .94; CL condition: *r* = .11; *p* = .57).

#### Time course and latency analysis

**Figure 3:**
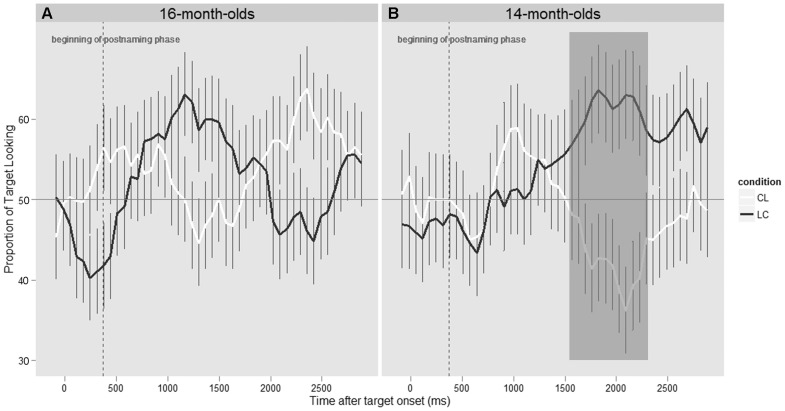
Time course of infants’ target looking behavior during the postnaming phase for LC words (black) and CL words (white) for both 16-month-old infants (Experiment 1, Fig. 3A) and 14-month-old infants (Experiment 2, Fig. 3B). The shaded box indicates the time period in which significant differences between the LC and CL conditions were found.

Since the analyses on the proportion of target looking failed to reveal differences in performance for the LC and CL words, we further looked for such differences between conditions in exploring the data of the postnaming phase in terms of its time course and infants’ latency to shift from the distractor to the target after naming the target (as done in e.g., [62], [75]–[76]). The time course analysis on looking behavior during the postnaming phase is plotted in Figure 3a (plotted for every 66 ms). While visual inspection of the time course suggests that LC words might be recognized earlier than CL words, statistical analyses, following von Holzen and Mani [76], did not reveal any significant differences between the two types of words, cluster *t* statistic = 5.65, Monte Carlo *p = *.68. (for additional details about the statistical procedure, see [77]).

Second, we examined the latency to shift from the distracter to the target, that is, the time needed to orient from the initially fixated distracter object to the target object after labeling (following the same analyses as done in e.g., [61]–[62]). These distracter-initial trials corresponded to 46.4% of all the trials. From these trials, we excluded trials in which infants shifted before the postnaming phase began as these saccades were probably programmed before the name of the target was processed (23%), or did not shift at all (7%) as well as outliers, that is values greater or smaller than two standard deviations from the mean (1%). Finally, only infants who provided both LC and CL data were included (N = 15, so only about half of the 28 infants tested). Mean latencies were of 775 ms (SD = 315 ms) for LC words, and of 782 ms (SD = 377 ms) for CL words. This was not significantly different, *t* (14) <1, therefore again failing to provide evidence of performance differences between the two conditions.

Taken together, the results of Experiment 1 show that 16-month-old infants are able to link both the LC and CL labels to the unfamiliar object referents presented in the present word learning task. No statistically reliable difference in performance could be found between the two conditions. This pattern of results is comparable with that of 20-month-olds who succeeded in learning both LC and CL words in the offline name-based categorization task used by Nazzi and Bertoncini [55]. It could thus be that the relative phonotactic probability of a sound sequence does not impact infants’ word learning at all, although phonotactic knowledge about the legality of sequences can constrain infant’s word learning by 17–20 months [41]. A second possibility however is that 16-month-olds are already too old to manifest such an effect in this task, thus that there is an earlier developmentally transient effect, as Nazzi and Bertoncini [55] have argued. To explore this possibility a group of younger infants aged 14 months was tested in Experiment 2, using the exact same multi-trial learning task as in Experiment 1.

## Experiment 2

### Materials and Methods

#### Participants

Twenty-eight full-term 14-month-old infants from French-speaking families were tested and included in the analysis (mean age = 14 months 10 days; range: 14 months 2 days –14 months 22 days; 10 girls, 18 boys). The data of eleven additional infants were not included in the analyses due to technical problems (n = 1), fussiness/crying (n = 2) or given that they did not fulfill the inclusion criteria (n = 8, see paragraph on data analysis for details). The age of these infants did not differ from the age of the included infants (mean age = 14 months 13 days, range: 14 months 3 days –14 months 22 days), and both female (N = 6) and male (N = 5) participants were excluded.

#### Stimuli, apparatus and procedure

The material, apparatus and procedure were the same as in Experiment 1.

### Data Analysis

The same data analysis and exclusion criteria were used as in Experiment 1. Three infants with less than 50% on-screen data (between 30% and 49%) were excluded from the analysis. 89 trials out of 256 (34.8%) were discarded because of containing more than 50% missing data and/or because of the infant displaying a strong object preference in the pre-naming phase. Five further infants were excluded because they had less than two analyzable trials per condition after trial exclusion. In the final sample (N = 28), each participant provided 5.96 trials on average. During each of the 30-sec learning phase, 14-month-old infants spent 6.6 s on average looking at the object and 12.6 s looking at the woman’s face. There were no difference in looking times at the objects for the LC (6.5 s) and CL (6.7 s) words.

Again, the receptive CDI score was taken as vocabulary measure.

### Results and Discussion

#### Label recognition measure: proportion of target looking

A repeated measures ANOVA on the proportion of target looking with phase (pre- vs. postnaming phase) and condition (LC vs. CL) as within-subject factors and gender (female vs. male) as between-subject factor revealed a marginal main effect of condition (*F*(1, 26) = 3.52, *p* = .07, *η_p_^2^* = .12) corresponding to a tendency for greater target looking in the LC condition (*M* = 54.22%, *SD* = 14.26%) compared to the CL condition (*M = *49.22%, *SD* = 15.09%). There was no significant effect of phase (*F*(1, 26 ) <1) but a significant interaction between phase and condition (*F*(1, 26 ) = 7.44, *p* = .01, *η_p_^2^* = .22). No effects including the factor gender reached significance (all *F* <1, except for the phase × gender interaction, *F*(1,26) = 1.25; *p* = .27). Comparisons within each condition revealed that while the proportion of target looking increased significantly across phases for the LC words (prenaming: *M = *50.35%, *SD* = 10.90%; postnaming: *M = *58.09%, *SD* = 16.26%; *t*(27) = 2.50, *p* = .02, Cohen’s *d* = .47), no effect of phase was found in the CL condition (prenaming: *M = *51.42%, *SD* = 13.34%; postnaming: *M = *47.01%, *SD* = 16.61%; *t*(27) = 1.40, *p*_ = _.19).

#### Comparing 14- and 16-month old infants

To evaluate whether the 14-month-old infants show a different looking behavior compared to the 16-month-old infants of Experiment 1, we conducted an ANOVA with all participants (N = 56), including age (14- vs. 16-month) and gender as between-subject factors and phase (pre- vs. postnaming) and condition (LC vs. CL) as within-subject factors. Of greatest importance, the 3-way interaction (phase × condition × age) was found to be significant, *F*(1,52) = 5.08, *p* = .03, *η_p_^2 = ^*.09. To specify this 3-way interaction, ANOVAs were conducted for each condition separately. For LC words, no effect involving age group reached significance (all F <1), establishing that learning of LC words is already present at 14 months of age and does not change with age. However, for CL words, age interacted significantly with phase, *F*(1,52) = 4.04, *p* = .04, *η_p_^2 = ^*.07. Single t-tests showed that at 14 months of age, there is no pre/postnaming increase in target looking for the CL words (*t*(27) = 1.40, *p*_ = _.19), but there is one at 16 months of age (*t*(27) = 2.32, *p = *.02, Cohen’s *d* = .44). This pattern of results demonstrates a developmental trend for CL words: From no novel word learning at 14 months to successful word learning at 16 months. Note again that gender did not have any significant (mediating) influence on word learning for both LC and CL words (all *F* <1). Therefore, the gender ratio difference between the two experiments (more boys than girls tested in Experiment 2) cannot explain the difference in word learning abilities across ages.

#### Correlation with vocabulary size

As in Experiment 1, the mean difference score of target looking between the pre- and postnaming phases ([% target looking in postnaming phase −% target looking prenaming phase]) was calculated for each participant and for both structures in order to examine the relationship with infants’ receptive vocabulary size. There was a trend towards a positive correlation between the CDI score and CL word recognition (*r = *.32; *p* = .09), that is there was a tendency for a link between the receptive vocabulary and the likelihood of learning CL words. This trend in the data was not observed for LC words (*r = *.01, *p = *.97).

#### Time course and latency analysis

As for Experiment 1, we also examined the data of the postnaming phase in terms of its time course and infants’ latency to shift from the distractor to the target after naming the target. The time course analysis on looking behavior during the postnaming phase is plotted in Figure 3b. Visual inspection of the time course suggests better performance for LC words than CL words, which is confirmed by the presence of significant differences between the LC and CL conditions between 1565 and 2290 ms after target word onset, cluster *t* statistic = 36.40, Monte Carlo *p* = .003 (as done by [76]).

Second, as in Experiment 1, switch latencies were calculated for those trials in which the participants fixated the distracter at the onset of the target word (49.1% of all the trials). From these trials, we excluded those trials in which infants shifted before the post-naming phase began (23%) or did not shift at all (9.6%) as well as outliers (4.8%). Only infants who provided both LC and CL data were included (N = 10, out of 28 infants). Latencies were of 970 ms (SD = 243 ms) for LC words and of 884 ms (SD = 329 ms) for CL words. This was not significantly different from each other, *t* (9) <1, providing no evidence of differences in LC and CL. However, it is important to note that the small sample size (N = 10) limits the power available for this analysis.

The results of Experiment 2 show that 14-month-old infants were able to link the most frequent phonotactic LC structures but not the less frequent CL words to the unfamiliar object referents presented in the word-learning task. These results are the first piece of evidence showing that infants’ word learning is impacted not only by knowledge about the phonotactic legality of sound patterns [41]–[42] but also by the relative phonotactic probability of a sound sequence at such a young age. Furthermore these results suggest that developmental changes in performance, since 14-month-olds could only learn the LC words, while the 16-month-olds tested in Experiment 1 and the 20-month-olds tested in Nazzi and Bertoncini [55] could learn both LC and CL words.

## General Discussion

The goal of the present study was to explore whether the relative phonotactic probability of a sound sequence in the native language has an impact on infants’ word learning. Accordingly, we tested 14- and 16-month-old French-learning infants using a multi-trial learning task involving eight pairs of pseudo-words consisting of phonotactically legal CVC strings paired with unfamiliar object referents. Half of the pseudo-words were Labial-Coronal sequences and the other half were Coronal-Labial sequences. These patterns vary in their relative frequency, LC sequences being much more frequent in the French lexicon than CL ones [11]. The results of Experiment 1 show that 16-month-old infants were able to associate LC and CL labels to unfamiliar object referents, with no significant difference in performance for the two types of labels (both in terms of mean looking times, time course analysis and latencies to shift from the distractor to the target). However, in Experiment 2, 14-month-old infants were only able to link the LC labels to the referents, showing that infants’ knowledge of their native language phonotactic patterns influences their word learning. Taken together, our results show that more frequent phonotactic word patterns were easier to learn, and were thus learned at an earlier age than infrequent phonotactic words. Therefore, the present findings contribute to prior evidence showing that native language phonotactic knowledge constrains word learning, showing that frequent versus infrequent legal sequences affect word learning by at least 14 months of age. Our results extend to novice word learners previous results on more expert children [22]–[27] and even adults [28]–[33], adding to phonotactic effects found at 12 months [42] and at 17–20 months [41].

Moreover, the present findings bring clear evidence showing developmental changes in learning CL words, but not LC words. At 14 months of age, infants were only able to associate the high-probability labels (LC) with the referent objects, while 16-month-olds were able to associate both frequent and infrequent phonotactic labels. These developmental changes can be explained by different hypotheses. The first possibility would be that phonotactic properties impact word learning, but only at the very beginning of this process. As vocabulary increases, the impact of phonotactics on word learning disappears. This possibility is not very plausible given the evidence reviewed earlier of phonotactic effects on word learning in older infants (18-month-olds, [41]), children [22]–[27], and even adults [28]–[33]. However, as discussed above, since different types of phonotactic regularities were explored in the present study (non-adjacent versus adjacent), it remains possible that they would follow different developmental trajectories, which would need to be directly assessed by studies exploring the two types of regularities at the same ages and using the same tasks.

A second possibility is that the developmental changes are due to “encoding facilitation,” as suggested by Saffran and Graf Estes [52], according to which words with a frequent phonotactic structure are easier to encode phonologically, and thus benefit from more available cognitive resources to be linked to referents. As vocabulary size increases, encoding proficiency improves, leading to a reduced phonotactic effect. This possibility is in line with our findings of better performance for the less frequent CL items at 16 compared to 14 months, and with the trend towards a positive correlation between receptive vocabulary size and CL word learning at 14 months. It is also compatible with the fact that looking times at the objects during training increased by about 3 seconds between the two ages (even though at both ages, looking times were identical for the two word structures). Therefore, while 6.6 s might be enough at 14 months to encode LC words, it might not be enough to encode CL words. However, at 16 months, 9.4 s of looking times at objects during training appear to be enough for infants to encode both word structures. Note that the proposal of reduced phonotactic effects does not mean that phonotactic effects fully disappear, as suggested by the fact that such effects are found even in expert word learners such as adults [28]–[33]. Therefore, it is likely that phonotactic effects could be found at all ages under conditions requesting high cognitive load. Regarding the 16-month-olds tested in Experiment 1, we predict that presenting fewer repetitions of each label, teaching more words at the same time, or using minimal contrasts (such as LC pat/bat or CL tub/dub, as done in [55]) might reveal phonotactic effects at 16 months. This possibility is in line with results obtained for children using tasks that required high cognitive load such as memory tasks requiring the recall of a list of words [27], word-learning tasks presenting fewer repetitions of each label [22], or repetition tasks presenting words with more syllables (from two to five syllables, [25]).

The present study also differs in two important other ways from the ones having previously found phonotactic effects. Specifically, our study exploits the presence of an LC bias in the French lexicon (the higher frequency of LC words over CL words) to explore effects of relative frequency rather than legality on the one hand, and of non-adjacent rather than adjacent phonotactic dependencies on the other hand. Regarding the first point, two other studies established that English-learning 12-month-olds [42] and 17–20-month-olds [41] can learn new label-object associations only if the labels are phonotactically legal in their native language. The present study extends the scope of the phonotactic effect from differences in legality to differences in relative frequency. This distinction is crucial. In the legality case, illegal sequences are sequences of sounds that are never heard as word-like units in the input, and that cannot be a word of the native language. So as infants become more proficient word learners, they should be less and less prone to learn words with phonotactically illegal structures. Although Graf Estes et al. [41] only tested one age group, correlation analyses showing that the size of the phonotactic effect increased with receptive vocabulary suggest that infants become more reluctant to learn words with illegal phonotactics. In the present case manipulating relative frequency, both high- and low-probability sequences occur as word-like units in the input, and both LC and CL stimuli were possible words in French. Therefore, infants need to be able to learn both types of words. Accordingly, the phonotactic effect we found corresponds to the fact that infants initially have difficulties learning the low-frequency words but become better learners of the low frequency words as they get older (from 14 to 16 months) and/or as their vocabulary increases (trend for a correlation between receptive vocabulary and CL word learning at 14 months). Hence, while the phonotactic effect becomes bigger with language acquisition when comparing the acquisition of legal versus illegal sequences [41], it becomes smaller when comparing the acquisition of high versus low frequency patterns.

This pattern of developmental reduction of the relative frequency phonotactic effect is congruent with previous results [55] that had failed to show such an LC/CL phonotactic effect in 20-month-old infants, who appeared to learn equally well LC and CL words. To explain this lack of effect, the authors had proposed that the task might not have been sensitive enough (infants had to provide an explicit reaching response to choose the target object), or infants were already too old. The present study suggests that task itself may not solely explain the lack of effect at 20 months, since no effect was found here at 16 months using a different task. However, only a direct comparison of the outcome of both tasks at the same age could confirm this possibility. On the other hand, our study shows that age must have contributed to the lack of results in Nazzi and Bertoncini [55], since the phonotactic effect that was clearly present at 14 months could not be found at 16 months. Taken together, both studies suggest that this relative frequency effect of phonotactic probability decreases, or becomes more subtle, as infants become better word learners.

The second important difference between the present study and previous ones on phonotactic effects is related to the kind of phonotactic pattern manipulated. Previous studies focused on adjacent properties of the specific items used as stimuli, in particular, on the frequency of clusters or adjacent diphones (e.g., [26], [28], [32]). On the contrary, the present study focused on the relative frequency of two structures differing in non-adjacent properties: the learning advantage was found for a structure (Labial-vowel-Coronal) that is more frequent in the target language than the other structure (Coronal-vowel-Labial), and the advantage is due to an asymmetry in the order of occurrence of the two non-adjacent consonants that are separated by a vowel. Therefore, the present study extends the scope of phonotactic effects on word learning from adjacent to non-adjacent dependencies, showing that the acquisition of both adjacent [2], [6], [78]–[79] and non-adjacent [11]–[12] phonotactic dependencies by 9/10 months of age impact later lexical acquisition.

Importantly though, it further appears that the present non-adjacent effect is not driven by knowledge regarding the relative frequency of the specific items used, since the stimuli were chosen so that the frequencies of all adjacent diphones and of the CVC items themselves were matched across the LC and CL structures (see [11], for similar effects in early perception). Hence the effect in Experiment 2 is likely to reflect the fact that 14-month-old infants are processing two abstract phonotactic structures/categories differently. If this is the case, the same word learning advantage for LC items should be found when presenting infants with specific LC and CL items chosen so that the LC items would have lower diphone and triphone frequencies than the CL items, a prediction that will have to be evaluated in future research. To further explore the relative importance of local properties versus non-adjacent frequencies, one could contrast LC sequences with sequences such as CC (Coronal-Coronal) sequences. Indeed, although coronal consonants are more frequent than labial ones overall in onset positions and in coda positions (c.f. [11]), LC combinations are more frequent than CC combinations in French. Based on the present findings, we predict that infants’ preferences should go to the LC items, those with higher non-adjacent frequencies, rather than the CC items, those with higher local properties (more frequent phonemes and more frequent adjacent frequencies).

At this point, we would like to discuss a couple of issues raised by the findings of the present study that could be explored in the future. The first issue relates to the kind of phonotactic patterns that can impact word learning. Given emerging data regarding the different roles that consonants and vowels play at different linguistic processing levels [20], [56], [80]–[81], it would be of interest to compare the impact of consonantal and vocalic phonotactic regularities on word learning. Second, based on the “encoding facilitation” hypothesis, the fact that LC word forms are easier to encode than CL word forms might facilitate not only their mapping to objects in word learning tasks, but might also facilitate their processing at other lexical or prelexical levels. One level at which such an effect could be found is on the ability to segment word forms from fluent speech. Such a facilitative segmentation effect has been found for other phonotactic regularities in infants [78]–[79] and adults [82]–[84]. This possibility has been recently investigated, and results show that LC sequences are easier to segment than CL sequences by French-learning 10-month-olds; however, by 13 months of age, French-learning infants are able to segment CL sequences as well [85]. Finally, the fact that the LC bias is not homogenously distributed across phonetic categories (given that it is not present in fricatives in French, c.f. [50]) opens the question about the level at which these non-adjacent acquisitions operate. A recent perceptual study [50] has found that French-learning infants have a preference for listening to LC sequences made up of plosives or nasals, but they show a preference for CL sequences made up of fricative sequences. These results are in line with the frequency results of the French lexicon showing an LC bias for plosives and nasals, but a CL tendency for fricatives [50]. Based on these results, we should expect a facilitation LC effect for plosives and nasals but not for fricatives in early word-acquisition. However, further studies are needed to confirm this possibility.

In conclusion, the present study provides new evidence showing that novel words with a frequent phonotactic structure can be learned more easily by 14-month-olds in the laboratory than novel words with a lower phonotactic structure probability. These findings show that prior knowledge about phonotactic regularities in the native language has an effect on early word learning, supporting theories according to which lexical acquisition is influenced by prior or parallel phonological acquisition. Furthermore these results show the existence of developmental changes in relative performance between 14 and 16 months of age, suggesting that effects of relative phonotactic frequency on word learning might only be observed in situations in which computational load is high.
